# A Comprehensive Review of Cardiac Tumors: Imaging, Pathology, Treatment, and Challenges in the Third Millennium

**DOI:** 10.3390/diagnostics15111390

**Published:** 2025-05-30

**Authors:** Andrea De Martino, Claudia Pattuzzi, Sara Garis, Francesca Bosco, Vittorio Maria Virgone, Antonio Salsano, Francesco Santini, Angela Pucci

**Affiliations:** 1 Division of Cardiac Surgery, Ospedale Policlinico San Martino, 16132 Genoa, Italyant.salsano@gmail.com (A.S.);; 2 Histopathology Department, Pisa University Hospital, 56100 Pisa, Italy; boscofrancesca10@gmail.com (F.B.); v.virgone@studenti.unipi.it (V.M.V.);; 3 DISC Department, University of Genoa, 16126 Genoa, Italy

**Keywords:** cardiac tumors, heart masses, pericardial tumors, histology, cardiac surgery, rare neoplasms, pseudotumors, cardiac metastases

## Abstract

Cardiac tumors represent rare neoplasms, but they include a very wide range of neoplasia—first primary benign and malignant cardiac tumors, then cardiac metastases, with these latter ones being far more common in adulthood. These diagnoses may be challenging because of frequently non-specific signs and symptoms; for example, their clinical management may be difficult because of the site and because of possible hemodynamic or arrhythmogenic consequences, independent from their biology. Cardiac tumors may be asymptomatic and incidentally diagnosed, or they may cause heart failure, life-threatening arrhythmias, or even sudden cardiac death. Although they may still represent a post-mortem finding, the evolution and the larger use of cardiac imaging tools, initially echocardiography, has progressively and significantly increased their in vivo detection. Magnetic resonance imaging and computed tomography may give crucial information as to the composition and localization of cardiac masses, useful for investigating them and for planning surgery. Histology is mandatory for the definite and differential diagnosis of the cardiac masses, for assessing predictive factors in malignancies, and for then establishing the appropriate management of patients. Modern techniques applied to histology, including immunohistochemistry and molecular biology, may be required to characterize cardiac tumors, to properly classify them and to assess predictive and/or prognostic markers. Surgical procedures, including minimally invasive surgery, have also dramatically evolved in the last decades, allowing adequate treatment in most cardiac tumors. Finally, biopsy may be useful in selected cases, particularly when radical surgery is not feasible, and histological diagnosis is fundamental for other possible therapeutic approaches. The scope of this review covers advancements in the imaging diagnosis, histology, and treatment of primary and secondary cardiac tumors.

## 1. Introduction

Cardiac tumors are rare neoplasms that can arise in any cardiac structure, including the myocardium, endocardium, valves, and pericardium [1]. Although they represent a rare clinical condition, with an estimated incidence of less than 0.3% of all tumors, they carry significant challenges in both diagnosis and management due to their varied nature and clinical consequences, including obstruction to blood flow, arrhythmia, valvular dysfunction, and embolic events [2]. They can also be asymptomatic, representing an incidental finding or even a post-mortem diagnosis [3].

Cardiac masses are constituted by primary tumors, i.e., neoplasms arising from cardiac structures such as the myocardium, great vessels, cardiac valves, or pericardium, and by secondary tumors, i.e., cardiac metastases, with a 20:1 secondary/primary ratio [4]. Diagnosis is mainly based on cardiac imaging, with 2-D echocardiography being crucial in most cases for first detecting cardiac masses [5]. Cardiac computed tomography (CT) or magnetic resonance imaging (MRI) may provide a more detailed evaluation of the tumor and its surrounding structures. Fluorodeoxyglucose positron emission tomography (FDG-PET) is helpful in staging malignancies and detecting metastases [2,5]. Histology is mandatory to establish the differential diagnosis between benign and malignant tumors, tumors and pseudo-tumors, and the histotypes of the neoplasm carrying on prognostic and therapeutic impact [6]. In challenging cases, endomyocardial or even (in selected cases) surgical biopsy of a cardiac mass may be helpful to investigate its possible neoplastic nature and its biological behavior, and to then make clinical decisions [3,7]. Surgical procedures have dramatically improved in the last decades, allowing radical surgery or the resection of cardiac masses in most cases, such as minimally invasive cardiac surgery in a few patients [8]. In select cases, cardiac transplantation or auto-transplantation can also be performed [9,10].

Finally, for malignant tumors, radiotherapy and chemotherapy, together with target therapies, are nowadays considered more and more frequently [11,12].

The present paper reviews the types, the diagnostic methods, and the management strategies for cardiac neoplasms with a focus on advances in diagnostic tools and treatment modalities.

## 2. Primary Benign Cardiac Tumors

Primary cardiac tumors originate from cardiac structures and are mostly benign, but they remain a significant cause of morbidity and mortality because of their biology and the difficulties in getting radical excision [6,9,13]. Cardiac imaging and histology are essential for their diagnosis. The increasing number of 2D-echocardiography procedures has a significant impact on their in-vivo and sometimes incidental detection, whereas histology is mandatory for their differential diagnosis and nowadays for possibly assessing predictive markers [14,15] (Table 1).

**a. Cardiac myxomas** represent the most common primary cardiac tumors (accounting for approximately 50% of cases). They are benign tumors, typically localized into the left atrium, though they can occur anywhere within the heart. The mean age of patients is 50 years, and they are slightly more frequent in women (2/1 ratio), but they can occur in pediatric age and in young patients, particularly in presence of a genetic predisposing condition called Carney’s complex (CNC) that is related to mutations of the PRKAR1A gene in at least 70% of the affected patients [16,17,18,19]. This rare genetic disorder can be found in 10% cases of cardiac myxomas, and it is associated with one of the multiple endocrine neoplasia (MEN) syndromes. In CNC, multiple and/or recurring atrial myxomas can occur, together with skin lesions (nevi, ephelides, cutaneous myxomas), other neoplasms (e.g., myxoid neurofibromas, large cell calcifying Sertoli cell tumors, thyroid tumors), and endocrine disorders, such as primary pigmented nodular adrenocortical disease (PPNAD) and acromegaly [20,21].

**Figure 1:**
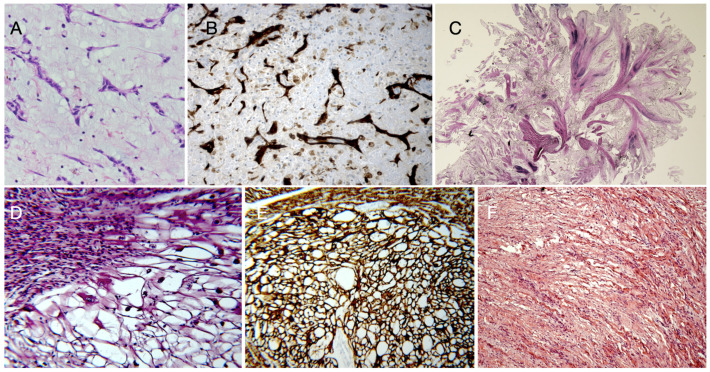
**Cardiac myxoma** is histologically characterized by polygonal, so-called lepidic cells into a myxoid stroma, often around small blood vessels (**A**); the cardiac myxoma cells show intense immunostaining for Calretinin (**B**). **Papillary fibroelastoma** has avascular papillary fronds, with a central fibro-elastotic stalk, surrounded by a layer of typical endothelial cells (**C**). Cardiac **rhabdomyoma** is characterized by large and vacuolated (**D**), desmin-positive (**E**) cells (so-called spider cells). **Fibroma** shows scattered fibroblasts, appearing as thin spindle cells lacking cytologic atypia and mitotic activity, in a dense collagen background (**F**). Hematoxylin and Eosin staining (**A**,**D**,**F**); immuno-peroxidase technique with hematoxylin counterstaining (**B**,**E**); Weigert staining for elastic fibers (in black; **C**). Original magnification: 10× (**A**,**B**,**D**,**E**) and 2× (**C**,**F**).

Cardiac myxomas are often asymptomatic, but they can also present with syncope, dyspnea, fatigue (depending mostly on blood flow obstruction), or arrhythmias that are potentially life-threatening and even may cause sudden death. Myxomas can be constituted by a polypoid mass with a smooth surface or by papillary forms, and they have a gelatinous consistency that can lead (more frequently in papillary forms) to embolic events and stroke if fragments dislodge [22]. The main histological characteristic of myxomas is the presence of scattered stellate or polygonal (lepidic) cells with an eosinophilic cytoplasm; oval or round nuclei; arranged singly, in cords, or around a vascular lumen into a myxoid stroma (Figure 1). They are considered to originate from multipotent mesenchymal cells and show immunoreactivity for Calretinin, S100, NSE, and variable expression of endothelial marker CD31 [4,16,23]. Hemorrhage, calcifications (including Gamma-Gandy bodies), and inflammatory infiltrates may be variably present. Even glandular structures have been rarely described, likely representing foregut rests that must be differentiated from metastatic adenocarcinoma [24].

The main differential diagnoses are represented by organized thrombi and myxoid sarcomas, with the cardiac myxomas showing no mitotic activity or tumor necrosis [25].

**b. Papillary fibroelastomas**, although representing rare tumors, with an incidence of less than 0.1%, are the second most common benign tumors in the heart in adults, and they are usually found on valve surfaces. Although usually asymptomatic, they can occasionally cause systemic embolic events, including the coronary system with myocardial infarction, and represent an indication for surgery [25]. Their most common location is the aortic valve (from 35% to 63%), with other valves commonly involved including the mitral (from 9% to 55%), tricuspid (6–15%), and pulmonary (from 0.5% to 8%) valve. These tumors are usually small (0.2–5 cm), round, and whitish, with a soft consistency and a gelatinous or villous appearance. A short stalk connects the neoplasm to the endocardium surface and gives the lesion a certain mobility (sea anemone-like structure) [26]. Histologically, papillary fibroelastomas are characterized by avascular papillary fronds, with a central fibro-elastotic stalk, surrounded by a layer of typical endothelial cells. The stalk contains elastic fibers, collagen, and mucopolysaccharides [4] (Figure 1). Occasionally, a superficial thrombus adhering to the fronds is found.

**c. Hemangiomas** arise from the endocardium, myocardium, or pericardium, in the ventricular free wall or in the right atrium. Most cardiac hemangiomas are asymptomatic and incidentally diagnosed. Otherwise, they may present with dyspnea, palpitations, murmur, thromboembolic events, or (in pericardial tumors) even hemopericardium and tamponade [4,27]. Congenital hemangiomas, frequently associated with pericardial effusion, may be diagnosed in utero. The congenital type of cardiac hemangioma has been rarely reported in Klippel–Trénaunay syndrome. Age ranges vary from the neonatal period to elderly. Approximately 10% of cases occur in children, and there is a slight male predominance. Usually, the tumors are single nodules; the endoluminal lesions (80% of cases) can be sessile or polypoid. Histologically, they are classified as capillary, cavernous, and arteriovenous, with the most frequent type being cavernous hemangiomas that exhibits large, dilated vascular spaces. Capillary hemangiomas consist of small, thin-walled vessels arising from and clustered around a larger vessel. Infantile capillary hemangiomas may show mitoses and express GLUT1 cavernous types, often situated within the myocardium itself. The arteriovenous types are characterized by arterial and venous structures; adipose tissue and collagen may be inter-mixed.

**d. Fibroma** represents another benign, but far less frequent cardiac tumor. It can cause significant issues depending on its size and location, such as ventricular arrhythmias, valve dysfunction, or severe obstruction of blood flow. Fibroma is more common in pediatric age, it may affect the ventricular myocardium appearing as a white and consistent mass; it is histologically characterized by scattered fibroblasts, appearing as thin spindle cells (sometimes with dystrophic calcifications) lacking cytologic atypia and mitotic activity, in a dense collagen background (Figure 1). They do not show a fibrous capsule and, although biologically benign, they may diffusely infiltrate the myocardium becoming unresectable, causing heart failure or severe arrhythmia, thus representing a possible life-threatening tumor for which cardiac transplantation may be considered [27].

**e. Lipoma** is composed of mature adipose tissue and can originate from the sub-endocardium (50%), sub-epicardium (25%), or myocardium (25%) [4,28]. Cardiac lipoma is more frequently located in the left ventricle or in the right atrium. It may occur at any age, from fetal period to elderly patients, although most lipomas occur in the 40–70 age group. It consists of a well-delimited mass composed by uniform adipocytes and may have thin fibrous septa with no cell atypia or mitosis. It must be differentiated from the lipomatous hypertrophy that is mainly found in the interatrial septum (sparing the fossa ovalis-Lipomatous atrial septal hypertrophy (LASH)) and representing an excessive fat deposition in advanced age or associated to obesity, and from the left ventricle lipomatous metaplasia that may be found in large myocardial infarction scars [29].

**f. Rhabdomyomas** represent hamartomas rather than actual neoplasms. They are detected between the 20th and the 30th gestational week and, in most cases, spontaneously regress during the third trimester of pregnancy [2]. Although rare, they represent about 45% of pediatric cardiac tumors and are the prevalent primary cardiac tumors in the pediatric population under 1 year of age. They may involve any cardiac chamber and are strongly arrhythmogenic. Rhabdomyomas can occur in association with genetic syndromes such as Tuberous Sclerosis Complex (TSC), which is a rare neurocutaneous disorder with an estimated prevalence ranging between 1/20-25,000 and 1/100,000. TSC is due to mutations in either TSC1 (9q34) or TSC2 (16p13.3) genes, which encode proteins indirectly inhibiting the mTOR pathway, with the mTOR excess causing increased cell growth and proliferation, as well as disproportionate glutamate activity, leading to disrupted synaptic plasticity. TSC clinical expression is variable because of mosaicism and/or genetic-epigenetic modifiers; it is characterized by hamartomas involving the skin, brain (cortical dysplasia or tubers, subependymal nodules, subependymal giant cell astrocytoma or SEGA), kidneys, lungs, eye, and heart (i.e., rhabdomyoma), and it is associated with neuropsychiatric disorders [6]. Multiple cardiac rhabdomyomas are common in TSC. Rhabdomyomas are usually well-circumscribed, firm, white-tan nodules within the myocardium. Rhabdomyomas originate from cardiac myocytes showing immature muscle differentiation, and are histologically characterized by large and vacuolated, desmin-positive cells (the so-called spider cells). The cytoplasm appears clear (Figure 1) on Hematoxylin and Eosin staining because of the abundant glycogen content [27].

**g. Benign/Low-Grade Tumors: Rare Histotypes**

Very rare histotypes have been described, sometimes representing masses that are exclusively found in the heart [4].

**Figure 2:**
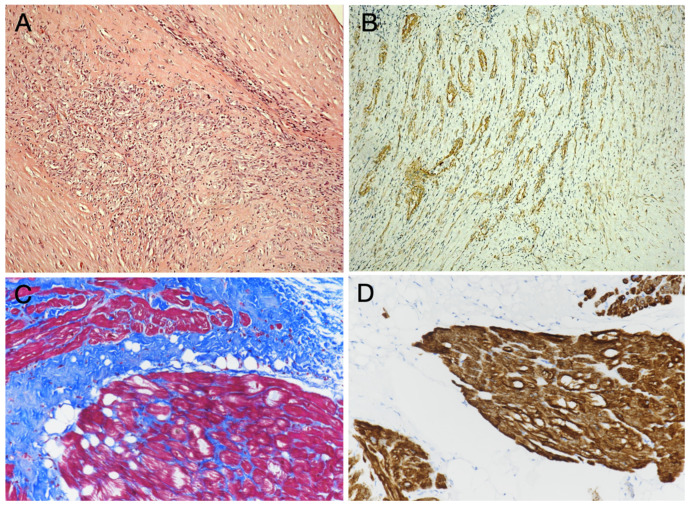
*Rare Benign Histotypes*. **Inflammatory myofibroblastic tumor** of the heart is characterized by a myofibroblastic proliferation (associated with limpho-plasmacellular infiltrates, (**A**) with smooth muscle actin immunoreactivity (**B**). **Hamartoma of mature cardiomyocytes** shows a proliferation of mature cardiomyocytes (**C**) with hypertrophy, partially delimited by connective and adipose tissue, with immunoreactivity for desmin (**D**). Hematoxylin and Eosin staining (**A**); immuno-peroxidase technique with Hematoxylin counterstaining (**B**,**D**); Masson’s Trichrome staining (**C**). Original magnification: 2× (**A**,**B**) and10× (**C**,**D**).

**Inflammatory myofibroblastic tumors (IMTs)** of the heart are very rare entities. They are endo-cavitary masses that may occur in any site (although more frequently reported into the right chambers) of the heart. IMT is considered a biologically benign/low-grade mesenchymal tumor, although it may have fatal consequences because of its location and recurrence; it is reported in 25% of extrapulmonary IMTs, in part related to anatomical site and resectability [30]. IMT appears as a whitish and usually polypoid mass with a smooth surface; it is mainly found in children and young adults. Histopathologic examination of cardiac IMT shows an endocardial-based cavitary mass composed of spindle cells (fibroblasts and myofibroblasts) with little or no atypia and no or very few mitoses (≤1 per 10 high-power fields), associated with inflammatory infiltrates, with mostly lymphocytes and plasmocytic cells in a fibrotic or myxoid stroma. The non-inflammatory cells are immunohistochemically positive for vimentin, calponin and smooth muscle actin, negative for CD34, myoD1, and pan-cytokeratin (Figure 2). Differently from its non-cardiac counterpart, cardiac IMT is often anaplastic lymphoma kinase-1 (*ALK*) negative, as shown by either immunohistochemistry or fluorescence in situ hybridization (FISH). *ALK* immunostaining patterns vary depending on the *ALK* fusion partner; *RANBP2*-*ALK* is associated with a nuclear membranous pattern, *RRBP1*-*ALK* with a perinuclear accentuated cytoplasmic pattern, and *CLTC*-*ALK* with a granular cytoplasmic pattern, whereas many other *ALK* fusion variants show a diffuse cytoplasmic pattern. *ALK*-negative IMTs may have a higher likelihood of metastasis, but *ALK* immunoreactivity does not apparently correlate with recurrence [31]. In *ALK*1-negative tumors, further genetic tests may be performed, searching for 2p23 locus clonal *ALK* gene rearrangements, *ALK* gene fusion with proto-oncogenes, and *ALK* overexpression. The molecular investigations may contribute to the definite diagnosis of such tumor, at least in controversial cases, in addition to carrying a prognostic significance.

**Hamartoma of mature cardiomyocytes (HMCM)** is extremely rare, and it is characterized by proliferation of mature cardiomyocytes with hypertrophy and sarcoplasmic vacuoles, partially delimited by connective and adipose tissue and immunoreactive for desmin and Myoglobin (Figure 2). Little is known about its pathogenesis; so far, it appears to be not associated with any known molecular or cytogenetic abnormalities. HMCM is often localized in the inter-ventricular septum (but it has also been reported in the atria) and may mimic hypertrophic cardiomyopathy; the definitive diagnosis requiring histology [32].

**Cardiac paragangliomas** are rare, hormonally active or inactive extra-adrenal tumors that arise from chromaffin cells of the sympathetic ganglia. They represent the 2–10% of all paragangliomas, the most frequent site is the left atrium, followed by the right atrium, aorto-pulmonary window, left ventricle, atrioventricular groove, inter-atrial septum, and right ventricle. They are more frequently found in young adults, and most cases show a benign behavior; a clinically malignant course can occur in up 10% of cases, in particular in extra-cardiac sites, with malignancy being assessed based on the presence of metastases [25,33,34]. Paragangliomas are characterized by a very high incidence of genetic predisposition and, as phaeochromocytomas, genetic and/or epigenetic alterations of ≥23 mutually exclusive genes may be responsible for tumor development. At least 17 are hereditary susceptibility genes, including *RET*, *NF1*, *VHL*, *TMEM127*, *SDHA*, *SDHB*, *SDHC*, *SDHD*, *SDHAF2*, *FH*, *MAX*, *EPAS1*, *DLST*, *MDH2*, *GOT2*, *SLC25A11*, and *DNMT3A*. Other genes that are not implicated in heredity but are somatically mutated in sporadic tumors include *HRAS*, *BRAF*, *SETD2*, *FGFR1*, *TP53*, *ATRX*, *ARNT*, *IDH1*, *H3-3A* (*H3F3A*), *MET*, and *CSDE1* [31]. Some mutated genes, especially *NF1*, *RET*, and *VHL*, are associated with both hereditary and sporadic tumors, whereas others, especially SDH genes, are exclusively hereditary. They may be diagnosed incidentally because frequently asymptomatic or may show symptoms related to mass effect or catecholamine release [34]. On gross examination, the tumors are soft, fleshy, highly vascular, broad-based, and usually tan or brown. Microscopically, they are identical to noncardiac paragangliomas, constituted by large and polygonal, epithelioid cells organized in nests or trabeculae with abundant granular eosinophilic cytoplasm, delimited by so-called sustentacular cells and with a variable amount of sclerotic stroma. By immunohistochemistry, cardiac paragangliomas are positive for tyrosine hydroxylase, DBH, GATA3, and neuro-endrocrine markers Chromogranin and Synaptophysin, whereas the sustentacular cells are S-100 positive. So far, there is no histological marker to predict metastatic disease, although multiparameter scoring systems have been proposed, including GAPP score. Their prognosis depends not only on complete tumor resection, but also on the genetic profile, with germline *SDHB* mutations conferring the highest risk of metastasis.

**Glomus tumors** are a group of rare mesenchymal neoplasms constituting <2% of soft tissue tumors [31]. They arise from smooth muscle cells of glomus bodies, which are specialized arteriovenous anastomoses, and may exceptionally affect the cardiac chambers [7]. In the heart, they are extremely rare and have been reported in pericardium or within ventricular walls. They are constituted by nests of rounded medium-sized cells with regular ovoid nuclei, lightly eosinophilic cytoplasm, and well-demarcated borders, around dilated vessels without necrosis, cell atypia, or mitotic activity (Figure 1). At immunohistochemistry, cells display strong reactivity for markers of smooth muscle cells such as alpha smooth muscle actin and caldesmon.

**h. Pseudotumors**

They represent cardiac non-neoplastic masses that may mimic cardiac tumors.

**Figure 3:**
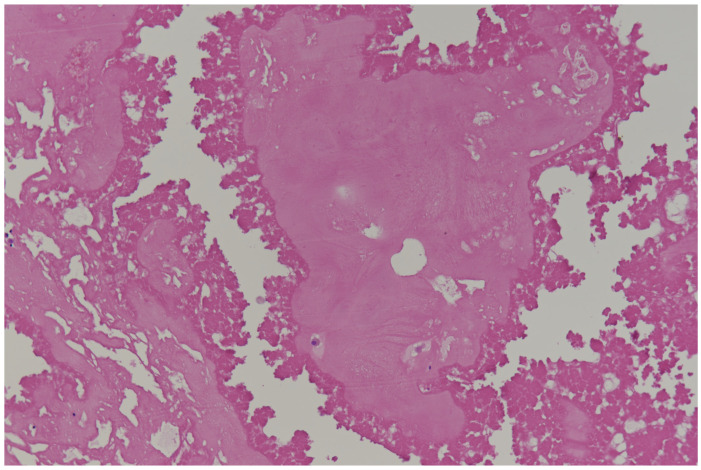
**Calcified amorphous tumor (CAT)** of the heart is composed of nodular calcium deposits within fibrinous material. Hematoxylin and Eosin staining. Original magnification: 2×.

**Calcified amorphous tumors (CATs)** of the heart are rare benign intracavitary masses of limited dimensions (maximum diameter measuring up to 1.5–2 cm in most cases) and composed of nodular calcium deposits within fibrinous material (Figure 3). The most common locations are the left ventricle and the mitral valve, but it may occur in any chamber of the heart. Pathogenesis is unknown, but it is frequently associated to valve diseases, particularly mitral annular calcification, end-stage renal disease, diabetes mellitus, and coronary artery disease [35].

**MICE or mesothelial/monocytic incidental cardiac excrescence** represents a rare entity, composed of a haphazard mixture of histiocytes, mesothelial cells, fibrin, adipocytes, and inflammatory cells without a vascular network or supporting stroma [36]. To date, its etiology has remained unclear. It is usually detected in adults and the elderly, and it is localized to the left atrium, mitral valve, pericardial sac, and right ventricle; however, it may also be localized to the right atrium, ascending aorta, right appendage, left atrioventricular groove and celiac artery. They must be differentiated from neoplasms or metastatic adenocarcinoma. 

**Blood cysts of cardiac valves** are seen in newborns and infants, and very rarely in adults. In most cases, they are incidental findings, but they may be associated to cardiac or systemic complications [37]. At histology, the cyst wall is composed mainly by fibrous tissue, with the inner surface being lined by a thin layer of typical endothelial cells.

## 3. Primary Malignant Cardiac Tumors

Primary malignant cardiac tumors (PMCTs) are rare neoplasms; they carry a poor prognosis due to their aggressive nature and metastatic potential [38,39]. The most frequent PMCTs are represented by sarcomas, followed by lymphomas, then by pericardial mesotheliomas; these latter ones are far less frequent than pleural mesotheliomas or may represent the extension of pleural mesothelioma to the nearby pericardium [40].

They are codified according to the WHO classification of soft tissue tumors and lymphoproliferative diseases, respectively [31,41]. Because of frequent late diagnoses and/or of difficulties in radical excision and treatment, they still carry a dismal prognosis, although therapeutic improvements are underway [39].

**Figure 4:**
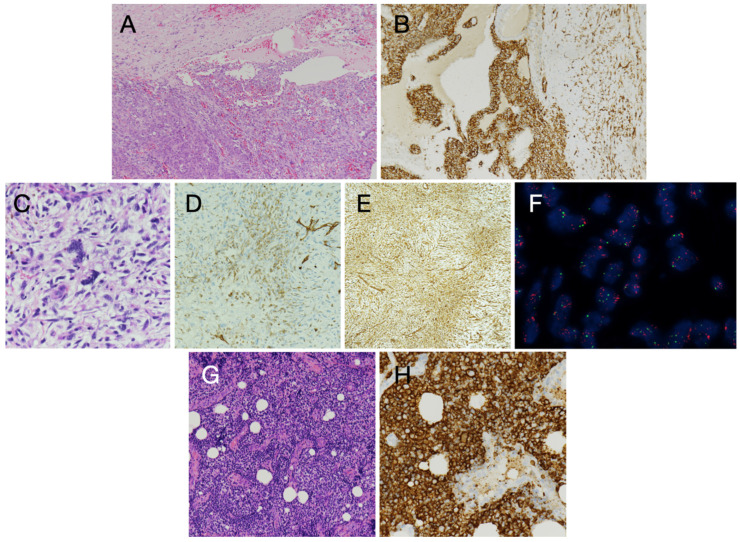
**Malignant primary cardiac tumors. Cardiac angiosarcoma** consists of irregularly shaped vascular channels lined by atypical endothelial cells (**A**) that show CD31immunoreactivity (**B**). Intimal sarcoma is mainly composed of highly atypical cells with nuclear pleomorphism and a high mitotic count (**C**). By immunohistochemistry, it shows focal positivity for CD31 (**D**) and more diffuse immunoreactivity for Vimentin (**E**). Fluorescence in situ hybridization (FISH) analysis shows MDM2 gene amplification (**F**) with several MDM2 gene red dot-like signals scattered over the nuclei. **Cardiac diffuse large B-cell lymphoma** (DLBCL) shows a diffuse myocardial proliferation (**G**) of CD20+ (**H**) B Lymphocytes. Hematoxylin and Eosin staining (**A**,**C**,**G**); Immuno-peroxidase technique with Hematoxylin counterstaining (**B**,**D**,**E**,**H**); FISH for MDM2 gene (**F**). Original magnification: 10× (**A**–**E**,**G**,**H**) and 100× (**F**).

**a. Angiosarcoma** is the most common sarcoma in adulthood, originating from endothelial vascular cells [6]. At clinical onset, it often shows symptoms of heart failure, due to tumor invasion of cardiac structures, and distant metastases. The right atrium is the most common site of origin, with a large, poorly circumscribed hemorrhagic and necrotic mass [2]. The infiltrative growth pattern makes the radical excision difficult by surgery [39]. By histology, these tumors consist of irregularly shaped vascular channels lined by atypical endothelial cells that show CD31 and ERG1 positivity by immunohistochemistry (Figure 4). The presence of poorly formed vascular spaces, lined by atypical, mitotically active, and highly proliferating cells (as shown by Ki-67/MIB-1 immunostaining for the proliferation index), are key features to distinguish angiosarcomas from benign vascular tumors. In poorly differentiated angiosarcomas, the neoplastic cells may be either spindle-shaped, rounded (epithelioid), or a combination, and immunohistochemistry for CD31 and ERG1 endothelial markers is crucial for the diagnosis [31].

**b. Rhabdomyosarcoma** is a rare, aggressive malignant tumor arising from striated muscle [41]. Although it accounts for only 4–7% of cardiac sarcomas, it represents the most common cardiac malignancy in infants and children, with a slight male predilection. At diagnosis, it is usually a bulky and infiltrative mass and frequently with distant metastases. This tumor may arise from the ventricular walls, but also from atrial (in adults, particularly) and valvular structures. It is made up of atypical skeletal muscle cells that may show nuclear pleomorphism (also with frequently multinucleated cells) and polygonal, spindled, or rhabdoid morphology, often a high mitotic count and presence of necrosis (Figure 2). They variably express desmin, MyoD1, and myogenin, depending upon the tumor differentiation grade [42].

**c. Intimal sarcoma** represents an undifferentiated sarcoma, characterized by MDM2 gene amplification [43]. It has been increasingly diagnosed in recent years, also because of improved histology tools. It has been mostly reported in middle-aged patients and in the elderly, but it has also been diagnosed in young adults. It may involve the major vessels, i.e., the pulmonary artery (mostly in women) and the aorta (in elderly males), as well as the pulmonary veins and the cardiac chambers [44]. Intimal sarcoma usually carries a dismal prognosis, related also to late diagnosis and/or impossible radical excision in most cases. The diagnosis of intimal sarcoma relies on histology, immunohistochemistry, and molecular pathology characterization. Histology shows a poorly differentiated tumor, mainly composed of highly atypical cells with nuclear pleomorphism and a high mitotic count (also >20/10 HPF). By immunohistochemistry, vimentin reactivity is usually associated with focal positivity for CD31. Immunohistochemical markers for epithelial cells, leukocytes, and melanocytes are all negative. Immunohistochemistry and FISH analysis show MDM2 gene amplification (Figure 4).

**d. Other sarcomas**, such as leiomyosarcoma, liposarcoma, and solitary fibrous tumor, may be found in cardiac chambers, both the atria and ventricles, or arising from the great vessels, the pulmonary veins or the pericardium [16,45]. Their definite diagnosis does require histology, including special techniques such as immunohistochemistry and molecular biology techniques, according to well-established diagnostic criteria [4,35].

**e. Primary cardiac lymphoma** is rare, accounting for 1–2% of the surgically resected heart tumors, with their incidence ranging between 0.15 and 1% at post-mortem examination. It is mainly represented by diffuse large B-cell lymphoma (Figure 4), although Burkitt lymphoma, low-grade B-cell lymphoma, and T-cell lymphoma have also been described [46]. It may involve any or all tissue layers and extend to the great arteries [41]. It may arise in the right chambers and may present with non-specific symptoms such as pericardial effusion and heart failure [2,3,46]. Primary cardiac lymphoma has been reported in adult patients, in either immuno-competent or immuno-compromised individuals. It may appear homogeneous, infiltrating white mass/masses, leading to wall thickening and restrictive physiology, or as nodular mass/masses intruding into the heart chambers.

## 4. Secondary Cardiac Tumors: Cardiac Metastases

Secondary cardiac tumors are far more common than primary cardiac tumors [1]. They are mostly represented by metastasis of lung adenocarcinoma and melanoma, but malignant tumors of any organ (e.g., breast and kidney carcinoma) may give cardiac metastases or neoplastic pericardial effusion [47]. Cardiac involvement by malignant neoplasia may occur via bloodstream or lymphatic spread, as well as by direct invasion. Clinical manifestations of secondary cardiac tumors may be subtle and nonspecific, including weight loss, fever, dyspnea, arrhythmia, heart failure, pericardial effusion, or cardiac tamponade due to pericardium involvement. Then, cardiac metastases are often diagnosed incidentally in patients presenting severe symptoms of heart failure, or even at post-mortem examination. The prognosis for patients with secondary cardiac tumors is poor, with heart involvement usually indicating advanced malignancy and a reduced overall survival rate. The heart may also be secondarily affected by lymphomas [38].

## 5. Clinical Presentation and Diagnosis of Cardiac Tumors

Clinical presentation of cardiac tumors can widely vary, depending upon the tumor type, size, location, and rate of growth [1,13,16]. Some tumors may be asymptomatic and detected incidentally during imaging for unrelated issues [39]. Furthermore, cardiac masses may show unexpected symptoms or debut with unexpected cardiac death [3]. Common symptoms can be summarized as follows:

- heart failure, caused by obstruction, valve dysfunction, or direct invasion of the myocardium;
- embolic events: tumors like myxomas can fragment and cause embolism, leading to stroke, organ infarction, limb ischemia, or pulmonary obstruction (right-sided);
- arrhythmias and ECG abnormalities, due to the disruption of normal electrical conduction, especially in case of intramural masses; patients may present ventricular tachycardia, left or right ventricular hypertrophy or atrioventricular block;
- chest pain that may occur due to direct tumor invasion or associated pericardial inflammation;
- syncope, often related to obstruction of blood flow or arrhythmias;
- symptoms related to pericardial effusion.

Other symptoms include fatigue, cough, fever, arthralgia, myalgia, weight loss, and erythematous rash, and laboratory findings are non-specific (anemia, increased erythrocyte sedimentation rate, increased level of C-reactive protein, and gamma globulin).

The diagnosis of cardiac tumors requires a combination of clinical evaluation and multimodal imaging studies, with the definite response relying on histology [16] (Table 2).

**a. Echocardiography** is often the first-line imaging tool for detecting cardiac masses, as it has high sensitivity and specificity. It represents the cornerstone of initial diagnosis due to its accessibility and non-invasive nature; it can provide valuable data on the size, location, and motion of the tumor. Otherwise, there are some findings suggestive of malignancy, such as the involvement of more than one cardiac chamber, multiple masses, location outside of left atrium, pericardial effusion, or an extension to the mediastinum or great vessels [48].

Transthoracic echocardiography (TTE) is non-invasive, widely available, provides real-time visualization of the heart, and plays a pivotal role in the diagnosis and assessment of cardiac tumors and their hemodynamic effects. The 2019 ACC/AHA/ASE Guidelines provide a flow-chart, based on the lesion identification, quantification of tumor size and mobility, functional impact and hemodynamics. It is important to assess the tumor’s attachment point and potential impact on adjacent structures such as the atrioventricular valves or septum and differentiating tumors from thrombi [49].

As to benign cardiac tumors, myxomas appear as echo-dense masses mostly attached to the interatrial septum. TTE can accurately reveal their size, shape (lobulated margins), attachment by a stalk to the atrial septum, and rage of motion, which is essential for pre-surgical planning.

Fibromas are typically small, brighter than surrounding structure, mobile, and may show signs of calcification. Lipomas are often well-defined and hypoechoic masses with no pedicle and motion. Papillary fibroelastomas often appear as small, homogeneous, mobile, and pedunculated masses on valve surfaces. On the other hand, malignancies have a great variety of morphological aspects, as they can grow into the pericardium, the myocardium, or directly into the chambers.

Accurate tumor measurement, especially in terms of diameter and volume, helps to assess their clinical significance, e.g., the size of myxomas correlates with embolic risk, but also allows for better surgical planning and post-operative monitoring.

Echocardiography is also crucial for evaluating the functional consequences of cardiac tumors. Tumors may obstruct blood flow, leading to symptoms such as syncope, dyspnea, or chest pain. Doppler echocardiography assesses blood flow velocities and any associated obstructive lesions. In the case of myxomas, for instance, Doppler may show restricted blood flow through the affected chamber, offering critical insight into the functional impact of the tumor. Tumors may interfere with normal valve function, leading to regurgitation or stenosis, with TTE making it possible to assess the extent of valvular dysfunction caused by tumors.

**Figure 5:**
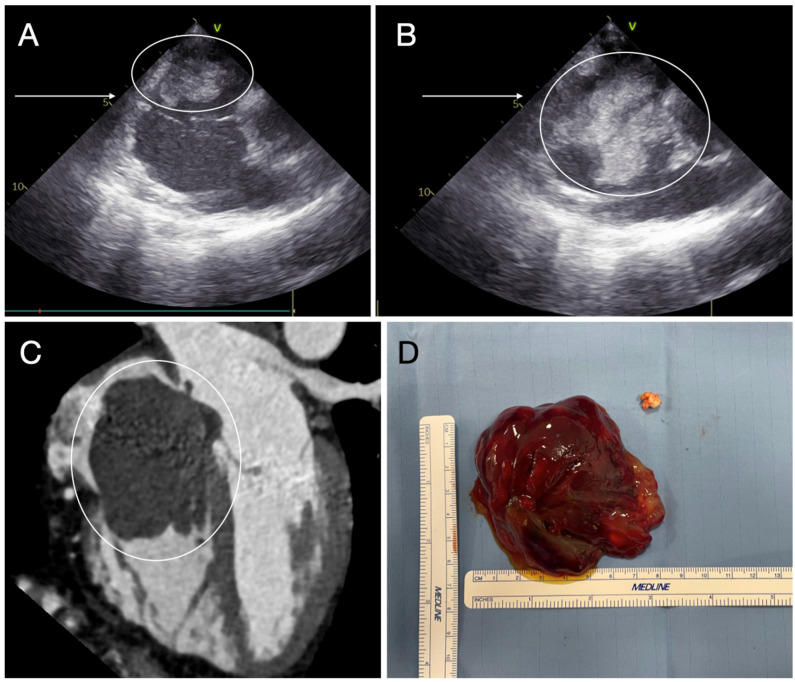
Transesophageal echocardiography shows a polypoid right-atrium mass (encircled) (**A**,**B**) adherent to interatrial septum by its pedicle (**A**), crossing the tricuspid valve (arrow) (**A**,**B**) in the diastole (**B**). Computed tomography reconstruction (**C**) confirms the characteristics of the mass (encircled) that shows the gross features of a cardiac myxoma after excision (**D**).

One of the challenges in cardiac imaging is distinguishing tumors from thrombi, particularly in patients with atrial fibrillation. Contrast-enhanced echocardiography can improve the visualization of tumors and differentiate them from thrombotic masses. Tumors typically have a more heterogeneous texture, while thrombi appear more uniform [48,49,50]. Recently, L’Angiocola and Donati provided a pragmatic approach to the identification of cardiac masses through echocardiography, highlighting the importance of high-resolution imaging in distinguishing between tumor types [51]. Transesophageal echocardiography (TEE) is more sensitive and useful for visualizing tumors in the atria and those located near the valves or septum, masses less than 5 mm, and fragments of the tumor in the left atrial appendage or other locations (Figure 5).

Finally, the potential of recurrence is typical for hemangiomas or cardiac myxomas in patients affected by CNC, and periodic echocardiography is recommended to examine for recurrence.

While echocardiography is an invaluable tool, it may be insufficient for characterizing tumors located in the posterior portions of the heart or in patients with poor acoustic window. In such cases, additional imaging modalities such as cardiac computed tomography (CT) or magnetic resonance imaging (MRI) may be necessary to provide a more detailed evaluation of the tumor and its surrounding structures (Table 3).

**b. CT** is largely used for the study of cardiac masses because of its high spatial and temporal resolution. Thanks to its imaging reconstruction capabilities and fast acquisition times, CT is a good diagnostic alternative, particularly in patients with known contraindications to MRI [39]. ECG-gated CT minimizes motion-related artifacts and gives superior spatial resolution for planning the surgical approach (Figure 5).

CT is useful to identify calcification, tumor angiogenesis, and thrombi, as well as to study the anatomy of chest, lung, and vascular structures. It can also exclude coronary artery disease. CT is used for tumor staging, showing possible metastases in case of suspect malignancy, and it is useful for the follow-up [52]. Disadvantages of this technique include exposition at radiation and contrast-related nephropathy. Furthermore, CT has a lower soft tissue resolution as compared to MRI. Several consensus statements supply guidelines for CT imaging as to the acquisition of imaging in the evaluation of cardiac masses. Protocols of post-contrast enhancement can help to differentiate between heterogeneous and homogeneous, between peripheral and central, etc. [53].

Very recently, the new technology of dual-energy CT (DECT) has given images useful tools to distinguish thrombi from neoplastic masses and to allow tissue characterization of cardiac masses based on iodine concentration [54].

**c. MRI** is a noninvasive multi-planar technique that is increasingly applied to cardiac masses evaluation, from intracavitary thrombi to primary or secondary intra-cardiac tumors, as well as to pericardial masses or pericardial extension of intracavitary ones. It is especially useful for assessing the characteristics of ventricular masses [55].

MRI has the best specificity in differential diagnosis: the multiple views and series can identify thrombi (by the lack of contrast enhancement and the presence of MRI blood products’ signature signal), which are the commonest “false” masses, but also endocarditic vegetations, lipomatous infiltration of the interatrial septum, crista terminalis in the right atrium, and hypertrophic interventricular septum.

**Figure 6:**
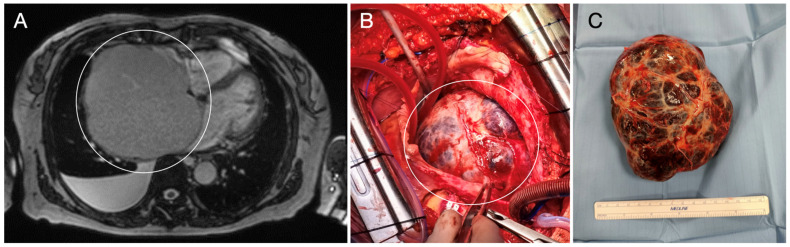
Magnetic resonance reconstruction shows a giant right atrial mass (encircled) with characteristics of a benign tumor (**A**); during surgery, the mass (encircled) is well-distinguishable from the chamber (**B**,**C**).

**Figure 7:**
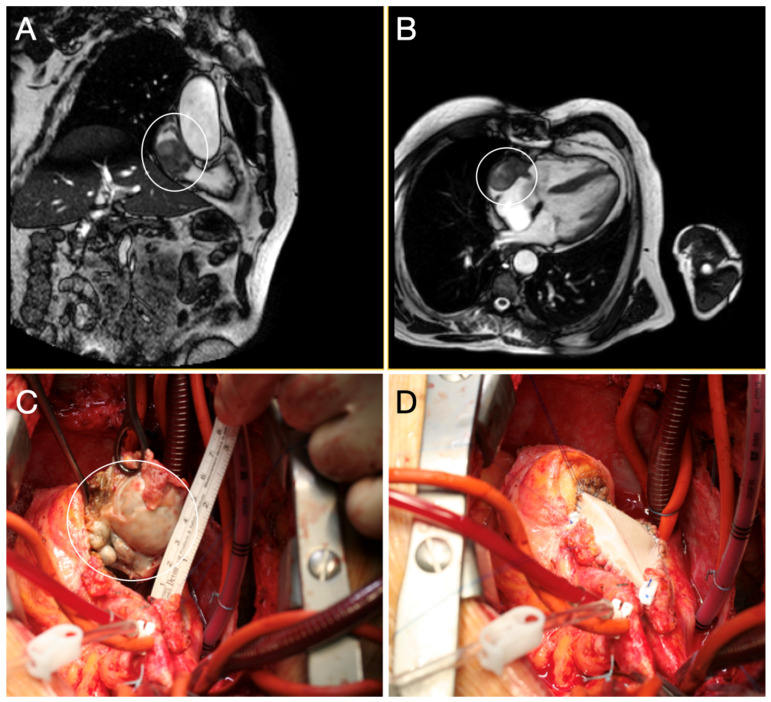
Magnetic resonance imaging (**A** coronal view, **B** axial view) shows an inhomogeneous (encircled) right atrium mass (6 × 4 × 3 cm), extending from the roof and free wall of the right atrium down to the inferior atriocaval junction, touching the outlet of the coronary sinus and tricuspid valve. The cardiac mass was surgically removed (**C**), with atrial-inferior vena cava junction reconstruction achieved by means of a bovine pericardial patch (**D**).

Furthermore, with peculiar protocols, it is the preferred technique in children when echocardiography poses the diagnostic suspect of cardiac masses, because it avoids radiation exposition [2,6]. MRI also gives information about extra-cardiac structures and their anatomy to plan surgical approach (Figure 6 and Figure 7). Serial MRIs are helpful for the follow-up to monitor tumor regression after surgery or chemotherapy [48].

Signal characteristics of this technique can help to define the characteristics of the mass such as necrosis areas, signs of active bleeding, calcifications, neovascularization, fatty infiltration [56]. The acquisition should provide T1-weighted black blood images with and without fat suppression, T2-weighted black blood images, first-pass myocardial perfusion imaging (to evaluate the vascularization), early gadolinium enhancement (to identify the thrombi), and late gadolinium enhancement (LGE). T1 and T2 mapping give instantaneous information about myocardial abnormalities and cardiac function [57].

MRI has some limitations, primarily the lower temporal resolution. Furthermore, MRI has long acquisition times and limited availability. Contraindications include claustrophobia and the presence of older and incompatible medical devices [55].

Nowadays, artificial intelligence can simplify and accelerate every step in the execution of MRI, including the optimization of protocols, image acquisition and reconstruction, postprocessing, image analysis, and the derivation of prognostic information. Furthermore, artificial intelligence could help in recovering high-spatial-resolution images from low-spatial-resolution observations containing noise [56,57].

**d. PET** offers an accurate evaluation of the metabolic activity of tumors using fluorodeoxyglucose (18F-FDG). FDG PET is helpful in staging malignancies and evaluating myocardial or pericardial involvement and detect metastases. It is also used for planning the therapy and to study the response. The different FDG uptake by tumor cells (which is quantified by the maximum standardized uptake value, the SUVmax) is useful for distinguishing benign from malignant tumors. However, some benign tumors (e.g., myxomas and hemangioma), infective vegetations, and a few inflammatory diseases can show high metabolic activity [57].

18F-FDG PET demonstrates its great diagnostic accuracy in differentiating benign from malignant cardiac masses with a sensitivity and specificity of 89.2% and 82.8%, respectively. Mean SUVmax values range from 1.1 to 5.3 for benign lesions and from 5.6 to 14.3 for malignant lesions, providing a valuable semi-quantitative parameter for clinical decision-making. This difference not only aids in distinguishing between benign and malignant masses but also offers prognostic insights [58]. Interestingly, higher SUVmax correlates with more aggressive disease behavior and poorer outcomes, highlighting the dual diagnostic and prognostic utility of PET imaging [59].

Limitations of this technology include dietary preparation and a radiation exposition. Furthermore, there are few studies that have suggested that PET imaging can add useful information to CT or MRI, improving the diagnostic setting in patients with cardiac masses, and PET remains a third-level diagnostic technique when CT and MRI images are inconclusive [60].

Recently, FDG PET has been combined with MRI in hybrid imaging systems. This 18F-FDG PET/MRI reduces false-positive results, but it has limited availability and high costs. Research is focused on the introduction of new imaging agents as specific tumor tracers, as those used in whole-body 99m Tc-HTOC (hydrazinonicotinamide-3-trypsin-octreotide) scintigraphy, that combine somatostatin receptor scintigraphy and CT images in detection of paragangliomas [61].

## 6. Biopsy of Cardiac Tumors

In selected cases, when non-invasive imaging does not yield to a diagnosis or it is anyhow difficult to make a clinical decision without a definite histological diagnosis, an endomyocardial or a surgical biopsy may be necessary to obtain adequate samples for histology. Moreover, when radical excision is not possible, excluding or assessing the malignancy of the mass is crucial, as well as for possible adjuvant treatments that might be considered based on histological diagnosis and characterization of the cardiac tumor [7,50]. Histology is indeed crucial for confirming the diagnosis and establishing the appropriate management of patients, addressing the clinical choices. However, biopsy requires experienced centers because it may carry risks, especially in highly vascular tumors [48].

## 7. Treatment and Prognosis

The treatment approach for cardiac tumors depends on their type and location, such as focusing on the histopathological evaluations that are crucial for determining the nature of the tumors and their potential for malignancy. It is critical to understand the histogenesis for effective management and prognosis [62].

**a. Surgery**. Surgical resection en bloc remains the treatment of choice for operable benign and symptomatic tumors with a generally favorable prognosis after complete removal. However, small and incidentally discovered left-sided masses often undergo surgical resection due to high embolic risk. For right-sided and asymptomatic benign cardiac tumors, in the absence of a patent *foramen ovale* or septal defects, strict echocardiographic follow-up can be employed [42].

Depending on the location, dimensions, and the probability of the tumor nature, surgical resection can be achieved by minimally invasive approaches, such as right mini-thoracotomy/endoscopy port-access, or by robotic technology [63,64]. Immediate disappearance of the tumor during surgery, confirmed by intraoperative TEE, coupled with regular follow-up echocardiograms to monitor recurrence, provides strong evidence for its effectiveness across different tumor locations and types. With an established safety profile, reduced resource utilization, less need for blood transfusions, and shorter hospital stays, particularly for high-risk patients like the elderly and obese individuals, minimally invasive approaches constitute an even more attractive option [65,66,67]. According to the literature, both thoracoscopic and robotic techniques offer advantages over traditional sternotomy in terms of safety and feasibility. However, robotic surgery might provide some additional benefits, like shorter operative time and length of stay, albeit potentially at a higher hospital cost [68,69].

For cardiac myxoma, surgical resection is the only effective therapy to be performed without delay to avoid embolization or obstruction to flow (Figure 5). In the case of papillary fibroelastoma of the heart valves, it is also recommended that the surgical resection is associated with valve reconstruction, if technically feasible. On the other hand, the surgical resection of lipomas, fibromas, and rhabdomyomas may not be recommended, unless in symptomatic cases.

Malignant tumors (particularly sarcomas) necessitate a tailored approach that may include surgical, chemotherapeutic, and radiotherapeutic interventions, although the benefit is uncertain; in select cases, an extended debulking, cardiac auto-transplantation (to improve exposure and radical excision), or orthotopic cardiac transplantation (in the absence of metastasis) may be considered.

Excision of tumors infiltrating the cardiac structures is often challenging due to difficult surgical exposure and the need for reconstruction by means of extensive pericardial patches (Figure 7) [8,70]. Cooley et al., in 1985, described a patient with a large pheochromocytoma requiring cardiac explantation and subsequent retransplantation for complete tumor removal and reconstruction [71].

Although complete resection is the treatment of choice for sarcomas, provided the patient has an acceptable performance status, most patients develop recurrent disease and eventually die, despite extensive surgical resection. In a series of 42 cardiac sarcomas treated at the Cleveland Clinic, the median survival was 25 months, with better outcomes in patients who received multimodality treatment compared with patients treated with surgery, radiation therapy, or chemotherapy only [72].

Lymphomas do not require surgical excision; their diagnosis and histological classification allow for established treatments. In cardiac lymphomas, a biopsy may represent the only tool for definite diagnosis and to address chemotherapy [2,3].

Primary pericardial mesotheliomas, due to late clinical presentation and extensive involvement of the pericardium and often of the underlying (ventricular) myocardium with a constrictive physiology, rarely benefits from surgical resection [73].

For secondary cardiac tumors, the treatment is generally directed against primary malignancy. Since these tumors indicate advanced disease, the prognosis is often poor, with high rates of relapse, and the focus is typically on palliative care and symptom management (diuretics, anticoagulation, and the management of arrhythmias) to improve quality of life [74]. Surgical therapy is limited to patients who suffer from recurrent pericardial effusion or cardiac tamponade [2,3,73]. New tools such as aspiration devices have been used in select cases to obtain a sample for histology and make diagnosis, to debulk neoplastic thrombi as palliative treatment, or as bridge to surgery in select cases [74,75].

In summary, surgery is the treatment of choice for cardiac tumors, as it allows complete excision of the lesion. However, there are alternatives for some types of tumors, such as those described previously, and it is controversial whether to adopt a conservative approach for cardiac tumors in asymptomatic patients. The decision-making process is sometimes difficult and should balance the risks of surgical treatment on the one hand with the risks of conservative treatment on the other. Conceptually, complete resection of the cardiac mass would be ideal. But not all tumors have a distinct capsule and can be invasive, rarely giving only the appearance of infiltrating healthy tissue. Therefore, the surgical dissection plane can be extensive and irregular, making reconstruction procedures of the heart and valvular structures necessary and complex, sometimes resulting in orthotopic heart transplantation [76]. Nevertheless, conservative therapy does not always make it possible to modify the (sometimes unknown) natural history of the tumor, as in the case of cardiac fibroma. Successful conservative management has been reported for cardiac hemangiomas with the use of corticosteroids, β-blockers, interferon-α, and anticancer drugs, such as vincristine and cyclophosphamide [77,78,79].

**b. Radiofrequency ablation** under TTE guidance has been recently described in poor candidates for surgery: through the puncture of the cardiac tumor via a percutaneous transthoracic approach, a biopsy is performed, and secondly, a radiofrequency electrode needle is introduced. This technique can be complicated by ventricular arrhythmia and pericardial effusion [80].

**b,c. Chemotherapy and radiotherapy** may be associated or not to surgery in PMCTs or in cardiac metastases. Doxorubicin, despite cardiotoxicity, remains one of the milestones for treatment of angiosarcomas, together with ifosfamide and trabectedin, especially used as adjuvant and lately neoadjuvant chemotherapy [81].

The most recent treatment strategy of primary cardiac sarcomas proposed by Chan et al. includes neoadjuvant chemotherapy until mass reduction and then surgical resection. Patients with metastatic disease or those whose tumors do not significantly respond to chemotherapy are generally not considered good candidates for surgical removal. Nonetheless, left-sided tumors can make neoadjuvant chemotherapy a risky or impossible option, with some chemotherapy drugs being cardiotoxic, potentially worsening heart function [82].

**d,e. Immunotherapy and targeted therapies**. Immunotherapy is based on immunomodulatory agents that stimulate the immune response against neoplasms, with early satisfactory results in selected cases such as advanced melanomas and sarcomas. Adjuvant treatment with anti-PD1 antibodies has been shown to effectively reduce the risk of recurrence in patients with melanoma. Genetically engineered T cells expressing cloned T cell receptors directed against sarcoma-associated antigens seem to be promising as adoptive immune therapy in sarcomas.

Targeted therapies, as in metastatic melanoma with BRAF mutations (usually in V600E), have the advantage of disrupting signaling pathways critical to tumor growth, such as those of tyrosine kinase inhibitors and anti-angiogenic agents [83,84,85].

Trials investigating anti-tyrosine kinase inhibitors (imatinib, pazopanib) in cardiac sarcomas have been conducted with good results, showing an acceptable rate of cardiovascular adverse events like hypertension, atrial fibrillation, and heart failure [86,87,88].

**f. Prognosis.** In a recent meta-analysis, the authors underlined the difference in mortality rates based on tumor type and the significant impact of surgical volume. The 5.90% short-term mortality and 2.55% long-term mortality rates provide a general overview, but the variability based on tumor subtype is key. The fact that low-volume centers (<5 cases annually) had more than double the mortality rate (>8%) compared to higher-volume centers strongly suggests that experience play a critical role in these complex surgeries, especially in the treatment of malignancies [89].

## 8. Conclusions

Cardiac tumors, albeit rare, are clinically significant because of their potential to cause severe cardiovascular complications. Early detection, using echocardiography and multimodality advanced imaging techniques, together with histological diagnosis and characterization, even using molecular methodologies whether appropriate, play a crucial role in determining the adequate treatment strategy. As a rule, management of cardiac tumors requires a multidisciplinary approach to guarantee a prompt diagnosis and the most appropriate treatment strategy. While benign tumors usually have favorable prognosis after surgical removal, malignant and metastatic cardiac tumors present significant challenges and are yet associated with poor outcomes.

Future directions in imaging diagnosis are essentially leading to increasingly accurate techniques to achieve very precise diagnosis before histology and characterize cardiac tumors more accurately, especially for surgery planning. The improvements in histological techniques, such as immunohistochemistry and molecular biology (FISH, PCR/reverse transcription polymerase chain reaction (RT-PCR), next-generation sequencing (NGS), etc.), will make the definite diagnosis and assessment of predictive or prognostic markers possible, aimed at possible target therapies or immunotherapies. Finally, while advancements in treatment of benign cardiac tumors are improving towards less invasive approaches, multimodal therapy and targeted therapies offer a glimpse of hope in the treatment of malignancies; as our understanding of the molecular underpinnings of these cancers grows, we can develop more precise and effective treatments. Integrating molecular diagnostics will be crucial for tailoring these therapies to individual patients.

## Figures and Tables

**Table 1:** Classification of cardiac tumors, modified from the WHO Classification of Tumours Editorial Board. Thoracic tumors [4].

**Benign tumors and tumor-like lesions** Papillary fibroelastoma Cardiac myxoma Cardiac fibroma Cardiac rhabdomyoma Adult cellular rhabdomyoma Cardiac lipoma and lipomatous hypertrophy of the atrial septum Lipomatous hamartoma of the atrioventricular valve Hamartoma of mature cardiac myocytes Mesenchymal cardiac hamartoma Cardiac hemangiomas Conduction system hamartoma Cystic tumor of the atrioventricular node	**Malignant tumors** Cardiac angiosarcoma Cardiac leiomyosarcoma Cardiac undifferentiated pleomorphic sarcoma Other sarcomas that may involve the heart **Hematolymphoid tumors** Cardiac diffuse large B-cell lymphoma Cardiac fibrin-associated diffuse large B-cell lymphoma

**Table 2:** Characteristics of tumors of the heart, modified from Maleszewski et al. [42]

Tumor Type	Age	Location	Syndromic Associations	Therapy/Prognosis
Cardiac myxoma	Adult	Atria	Carney complex	Surgical excision/good
Papillary fibroelastoma	Adult	Valves	–	Surgical excision/good
Hemangioma	Adult	Ventricles	–	Surgical excision/good
Fibroma	Infant	Ventricles	Gorlin syndrome	Imaging monitoring/good
Lipoma	Adult	Pericardium	Tuberous sclerosis	Imaging monitoring/good
Lipomatous hypertrophy of the atrial septum	Adult	Atria	–	Imaging monitoring/good
Rhabdomyoma	Infant	Ventricles	Tuberous sclerosis	Spontaneous regression/good
Inflammatory myofibroblastic tumor	Infant	Valves	–	Imaging monitoring ± surgical excision/variable
Paraganglioma	Adult	Atria	–	Surgical excision/variable
Hamartoma of mature cardiac myocytes	Adult	Ventricles	–	Imaging monitoring ± surgical excision/good
Glomus tumor	Adult	Ventricles/Pericardium	–	Surgical excision/good
Angiosarcoma	Adult	Atria	Li–Fraumeni syndrome	Surgical excision ± Chemotherapy/recurrence
Rhabdomyosarcoma				Surgical excision ± Chemotherapy/recurrence
Intimal sarcoma				Surgical excision ± Chemotherapy/recurrence
Lymphoma	Adult	Pericardium	–	Chemotherapy/variable
Metastasis	Adult	Ventricles	–	Chemotherapy/poor

**Table 3:** Tissue characterization and advantages/limitations by imaging modality, modified from Maleszewski et al. [42]. The sensitivity and the advantage of the techniques range from very low (−) to very high (++++).

Feature/Advantage	Echocardiography	MRI	CT
Calcium	+++	+	++++
Extracardiac extension	+	+++	++++
Fat	++	++++	+++
Mobility	++++	++	++
Thrombus	++	++++	+++
Coronary characterization	−	++	++++
Availability	++++	++	+++
Children	++++	++	+
Radiation	−	−	++

## Data Availability

No new data were created or analyzed in this study.

## Abbreviations

The following abbreviations are used in this manuscript:

CT	Computed tomography
MRI	Magnetic resonance imaging
FDG-PET	Fluorodeoxyglucose Positron emission tomography
CNC	Carney’s complex
MEN	Multiple endocrine neoplasia
PPDAP	Primary pigmented nodular adrenocortical disease
LASH	Lipomatous atrial septal hypertrophy
TSC	Tuberous sclerosis complex
SEGA	Subependymal giant cell astrocytoma
IMT	Inflammatory myofibroblastic tumor
ALK	Anaplastic lymphoma kinase
FISH	Fluorescence in situ hybridization
HMCM	Hamartoma of mature cardiomyocytes
CAT	Calcified amorphous tumor
MICE	Mesothelial/monocytic incidental cardiac excrescence
PMCT	Primary malignant cardiac tumor
HPF	High-power field
TTE	Transthoracic echocardiography
TEE	Transesophageal echocardiography
DECT	Dual-energy CT
LGE	Late gadolinium enhancement
SUV	Standardized uptake value
HTOC	Hydrazinonicotinamide-3-trypsin-octreotide
RT-PCR	Reverse-transcription polymerase chain reaction
NGS	Next-generation sequencing
